# Evaluation of the Antioxidant, Cytoprotective and Antityrosinase Effects of *Schisandra chinensis* Extracts and Their Applicability in Skin Care Product

**DOI:** 10.3390/molecules27248877

**Published:** 2022-12-14

**Authors:** Martyna Zagórska-Dziok, Magdalena Wójciak, Aleksandra Ziemlewska, Zofia Nizioł-Łukaszewska, Uliana Hoian, Katarzyna Klimczak, Dariusz Szczepanek, Ireneusz Sowa

**Affiliations:** 1 Department of Technology of Cosmetic and Pharmaceutical Products, Medical College, University of Information Technology and Management in Rzeszow, Kielnarowa 386a, 36-020 Tyczyn, Poland; 2 Department of Analytical Chemistry, Medical University of Lublin, Chodźki 4a, 20-093 Lublin, Poland; 3 Chair and Department of Neurosurgery and Paediatric Neurosurgery, Medical University of Lublin, 20-090 Lublin, Poland

**Keywords:** *Schisandra chinensis*, antioxidant activity, irritation potential, tyrosinase inhibitor, cosmetic safety

## Abstract

Plant extracts have been widely used for skin care for many centuries, and nowadays, they are commonly applied for the development and enrichment of new cosmetic preparations. The present study aimed the assessment of the biological activity of aqueous *Schisandra chinensis* extracts as a potential ingredient of skin care products. The aspects studied involved the ability to neutralize free radicals, impact on viability and metabolism of keratinocytes, as well as tyrosinase inhibitory potential. Our study showed that aqueous *S. chinensis* extracts have a positive effect on keratinocyte growth and have high antioxidant potential and strong tyrosinase inhibitory activity. UPLC-MS analysis revealed that three groups of phenolic compounds were predominant in the analyzed extract, including lignans, phenolic acids and flavonoids and protocatechiuc and p-coumaryl quinic acids were predominant. Moreover, microwave-assisted extraction, followed by heat reflux extraction, was the most effective for extracting polyphenols. Furthermore, a prototypical natural body washes gel formulation containing the previously prepared extracts was developed. The irritation potential and viscosity were assessed for each of the formulations. The study demonstrated that the addition of these extracts to body wash gel formulations has a positive effect on their quality and may contribute to a decrease in skin irritation. In summary, *S. chinensis* aqueous extracts can be seen as an innovative ingredient useful in the cosmetic and pharmaceutical industry.

## 1. Introduction

Plant extracts are currently one of the most numerous and most highly appreciated groups of ingredients used in the pharmaceutical and cosmetic industries. In addition to their potent antifungal, antibacterial and antiviral activity, they also exhibit strong antioxidant properties [1,2]. Moreover, they are commonly used in skin-lightening products formulated for sensitive and allergy-prone skin [3]. Active substances contained in plant extracts may contribute to a decrease in the irritation potential of cosmetic products. They may delay the process of decomposition of unsaturated lipids in the intercellular matrix and thus protect the skin against the impairment of the epidermal barrier, ultimately reducing the potential for skin irritations and improving microcirculation [4]. Plant extracts are valuable cosmetic additives for enhancing their quality and improving functionality [5,6]. It should also be mentioned that extracts have been shown to be much more effective than individual compounds because of synergistic interactions taking place between all chemical ingredients [7,8]. 

One of the plants containing plenty of active components with high potential as an ingredient of cosmetics is schisandra (*Schisandra chinensis* L.), used in traditional Chinese medicine for centuries. The main part of the plant harvested for therapeutic applications is the fruit which is rich in a wide range of active components. The main group of compounds found in schisandra fruit is dibenzyloctadienelignans, in amounts ranging from 7.2 to 19.2% DM [9]. In addition to lignan, schisandra fruit also contains phenolic acids, flavonoids, anthocyanins, organic acids, triterpenes (schintrilactone A, B), phytosterols (stigmasterol, β-sitosterol), essential oils or vitamins E and C [9,10,11]. Extracts derived from *S. chinensis* fruit have antioxidant, anti-allergic and anti-inflammatory properties. In addition, they show radiation-protective, anti-aging, soothing, and bleaching [11,12,13,14,15]. Multiple studies have demonstrated the anti-aging effects of *S. chinensis* and justified its classification as a plant with anti-aging and regenerative qualities. *S. chinensis* extract has also been shown to reduce the levels of inflammatory mediators, including histamine, IgM and IgE, which confirms the anti-allergic and soothing properties of the extract [14]. Anthocyanins and flavonoids (quercetin, rutinoside) contained in *S. chinensis* fruits seal the capillary walls, reduce their permeability and produce anti-allergic effects [15]. 

The type of solvent for extraction has a key impact on the chemical composition of the obtained product and, therefore, the biological properties. A mixture of water and the organic solvent is the most effective to isolate polar active compounds; however, organic additives should be avoided in the preparation of extract for skin care because they are toxic and such extracts are not suitable for direct usage as a cosmetic ingredient. Evaporation of organic solvent is required, and such an approach may result in unfavorable processes in extract including degradation because of elevated temperature. One-step solvent extraction is the most frequently used in the cosmetic industry and water is still the safest and ecologically friendly extrahent for cosmetic application. Water also allows the extraction of water-soluble components, which can be easily absorbed by the skin [16,17]. The main disadvantage of extraction using water is low effectiveness for isolation of secondary metabolites, which determine the activity of the extract. To enhance efficiency, the process may be assisted, e.g., by heating, microwave and ultrasound; however, it may alter the chemical composition and properties of the extract. 

The aim of the study was to determine the effect of assisted extraction techniques using water as extrahent on the content of active ingredients in the extract of *S. chinensis* fruits. The aspects studied also involve the biological potential of the products, including the ability to neutralize free radicals, impact cell growth and metabolism as well as tyrosinase inhibitory potential. Furthermore, a prototypical natural body washes gel formulation containing the previously prepared extracts was developed. The irritation potential and viscosity were assessed for each of the formulations.

## 2. Results 

In our study, water extracts from *S. chinensis* fruits were prepared using maceration with continuous stirring at room temperature (ME), elevated temperature (HAE) and extraction assisted by microwave (MAE) and ultrasound (UAE). For phytochemical analysis and biological experiments, non-evaporated extracts were used.

### 2.1. Phytochemical Composition of the Extracts and Quantitative Analysis of Main Constituents

**Figure 1:**
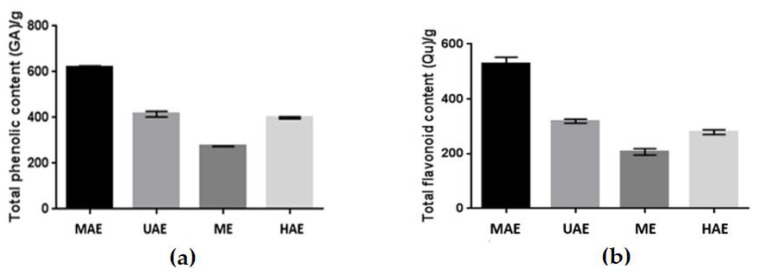
Total phenolic content (**a**) and total flavonoid content (**b**) in four types of aqueous extracts (UAE-ultrasound-assisted extraction; MAE–microwave-assisted extraction; ME–maceration with continuous stirring; HAE–heat-assisted extraction) of *Schisandra chinensis* fruits. Values are the mean of six replicate determinations (*n* = 6) ± SD.

Preliminary, total phenolic (TPC) and flavonoid (TFC) content were assessed as gallic acid and quercetin. The results are presented in Figure 1.

The phytochemical composition of the extracts was established using UHPLC-MS. The components were identified in negative and positive ionization modes based on mass data (M − H and M + H), fragmentation pattern and UV-Vis spectra (200–600 nm) and they were compared with standards when available. For other components, identification was based on literature data. Representative chromatograms are shown in Supplementary Material (Figure S1), and mass data are summarized in Table 1 and Table 2.

Three groups of phenolic compounds were predominant in the analyzed extract, including lignans, phenolic acids and flavonoids. Identified flavonoids belong to two groups. Quercetin derivatives: glucoside, galactoside, and rutinoside displayed common MS patterns with pseudomolecular ion characteristics for quercetin *m/z*-H 300. Kaempferol derivatives: rutinoside and glucoside showed pseudomolecular ion corresponding to aglycon form (*m/z*-H 285). Protocatechuic acid and p-coumaryl quinic acids (with parent ion *m/z*-H 337.09) were the predominant phenolic components of the extract. p-coumaryl quinic acid was present in the form of some isomers showing characteristic fragment ions: *m/z* 163 was typical for 3-*p*-coumaroylquinic acid, the fragment ion at *m/z* 173 indicated the substitution of quinic acid at position 4 (4-p-coumaroylquinic acid) and the last isomer showed the main peak at *m/z* 191 assigned to 5-pcoumaroylquinic acid [18]. Quinic acid–a commonly found component in the plant, was also identified. Moreover, two esters of caffeic and quinic acid (neochlorogenic and chlorogenic acid *m/z* 353.088) with pseudomolecular ions *m/z*-H 179 and 191 correspond to both components, respectively were also detected [19]. Among anthocyanin, one component at 19.40 min. With characteristic UV-Vis spectrum and maximum absorbance in the visible region has been observed. The ionization at positive mode showed parent ion at *m/z* +H 727.20886 (generated formula: C_32_H_39_O_19_^+^, Δppm 1.18) and additional pseudo molecular ion 287 corresponding to aglycon cyanidin and based on comparison with the literature [20] it has been identified as cyanidin 3-*O*-xylosyl-rutinoside. 

Lignans, because of lower polarity than flavonoids and polyphenolic acids, were separated using less polar eluents [18,21]. Moreover, they were ionizable both at negative and positive modes. Four main components have been identified, and among them, Schisandrol A was predominant. Detailed MS data were summarized in Table 2, and representative chromatograms and MS spectra were shown in Supplementary Material (Figures S2 and S3).

Representative UV-Vis spectra of all identified compounds were presented in Figure S4. 

**Table 2:** MS data of lignans identified in *S. chinensis* fruits extract at positive ionization mode.

TR (min.)	Observed Ion Mass [M + H]^+^/(Fragments)	ppm	Formula	Identified	Ref.
5.50	433.22431 (415, 455)	4.47	C_24_H_32_O_7_	Schisandrol A	[18,22]
7.10	417.19097 (399)	0.46	C_23_H_28_O_7_	Schisandrol B	[18,22]
9.80	501.24861 (401,483,523,539)	0.63	C_28_H_36_O_8_	Micrantherin A	[18]
11.30	530.25361 (431,548,569)	4.87	C_29_H_37_O_9_	Angeloylgomisin Q	[18]

The main identified compounds from phenolic acid and flavonoid groups were quantitatively analyzed and expressed as µg/mL of extract (Table 3). Because of a lack of standards, lignan content was compared based on peak area (Figure S5). 

### 2.2. Antioxidant Activity

**Figure 2:**
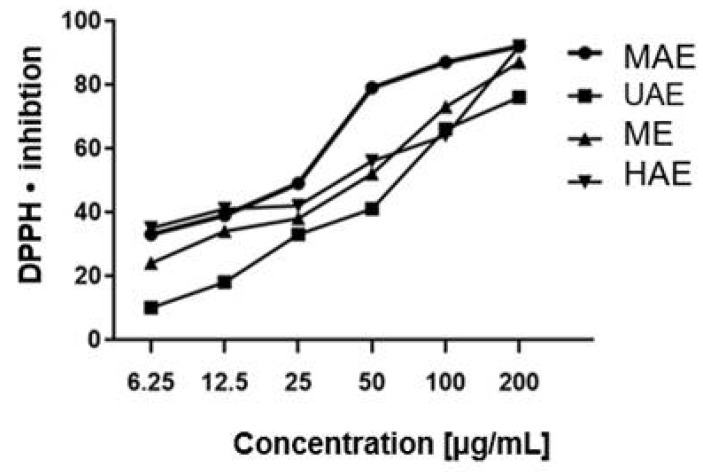
Antioxidant properties of *Schisandra chinensis* aqueous extract against DPPH free radical solutions. MAE–microwave-assisted extraction; UAE-ultrasound-assisted extraction; ME–maceration with continuous stirring; HAE–heat-assisted extraction. The concentration range of extracts is from 6.25 to 200 µg/mL. Values are the mean of three replicate determinations (*n* = 3).

The analyses showed the extracts under study to possess varying antioxidant properties. The extract obtained by the microwave method was characterized by the highest antioxidant capacity at a concentration ranging from 6.25 to 200 µg/mL. The lowest antioxidant capacity was shown for the ultrasound-treated extract: approximately 10% of DPPH inhibition at a concentration of 6.25 µg/mL. Free radical scavenging activity was found to increase gradually with increasing concentrations in all tested extracts (Figure 2).

**Figure 3:**
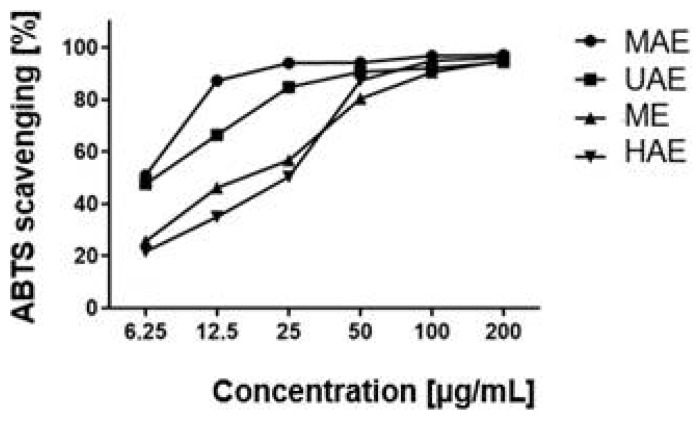
Antioxidant properties of *Schisandra chinensis* aqueous extract against ABTS solutions. UAE-ultrasound-assisted extraction; MAE–microwave-assisted extraction; ME–maceration with continuous stirring; HAE–heat-assisted extraction. The concentration range of extracts is from 6.25 to 200 µg/mL. Values are the mean of three replicate determinations (*n* = 3).

An analysis of the radical cation ABTS•+ also demonstrated strong antioxidant properties in the extract obtained by the microwave method. A marked decrease in antioxidant activity was shown for the ME and HAE extracts in the concentration range of 6.25–200 µg/mL. This indicated the weak antioxidant properties of these extracts toward the radical under study. For the extracts subjected to a high temperature and classic maceration, the scavenging of the radical ABTS•+ at different concentrations was at a similar level. At the concentration of 200 µg/mL, it was 95% ABTS scavenging and increased gradually along with increasing concentrations (Figure 3).

### 2.3. Cytotoxicity Assessment

**Figure 4:**
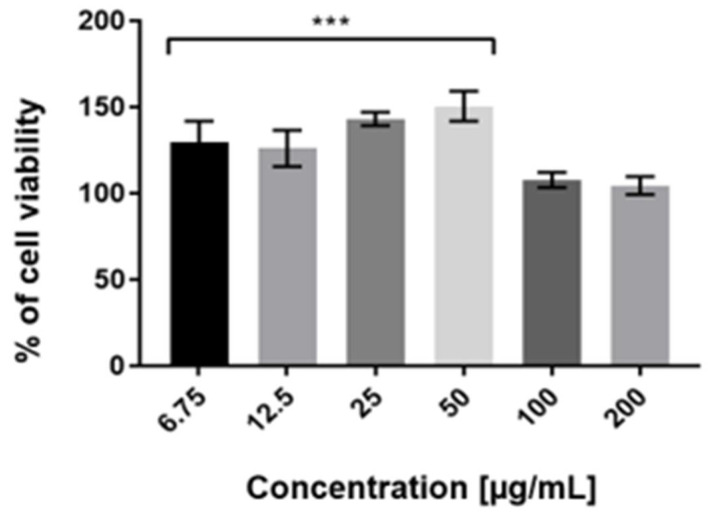
The effect of increasing concentrations of aqueous MAE *Schisandra chinensis* extracts (6.75, 12.5, 25, 50, 100, 200 µg/mL) on Neutral Red Dye uptake in cultured keratinocytes after 24 h of exposure. Data are the mean ± SD of three independent experiments, each of which consists of three replicates per treatment group. *** *p* < 0.001 versus the control (100%).

To assess the cytotoxicity of examined extracts against HaCaT cells in vitro, the Neutral Red (NR) uptake assay was used. The cytotoxicity is expressed as a concentration-dependent reduction of the uptake of NR after exposure to the analyzed compounds. Analysis of cytotoxicity against keratinocytes was carried out for all tested extracts (ME, UAE, HAE). However, the results obtained demonstrated that MAE *Schisandra chinensis* extract showed the most favorable proliferative activity in all tested concentrations (Figure 4). These results also correlate with its highest antioxidant potential relative to the other extractants used. After the treatment of keratinocytes with 50 µg/mL MAE extract, the number of living cells increased by up to 150% compared with the control group. The highest concentration of *Schisandra chinensis* extract (6.75–50 µg/mL) exhibited proliferative properties, but when the dose increased, the cell viability decreased. Our study demonstrated that the applied concentrations of MAE extract had not caused a reduction in the uptake of neutral red dye. This indicates that the extract (in the range of used dilutions) affects the maintenance of the pH gradient in HaCaT cells and determines the production of ATP at the expected level.

**Figure 5:**
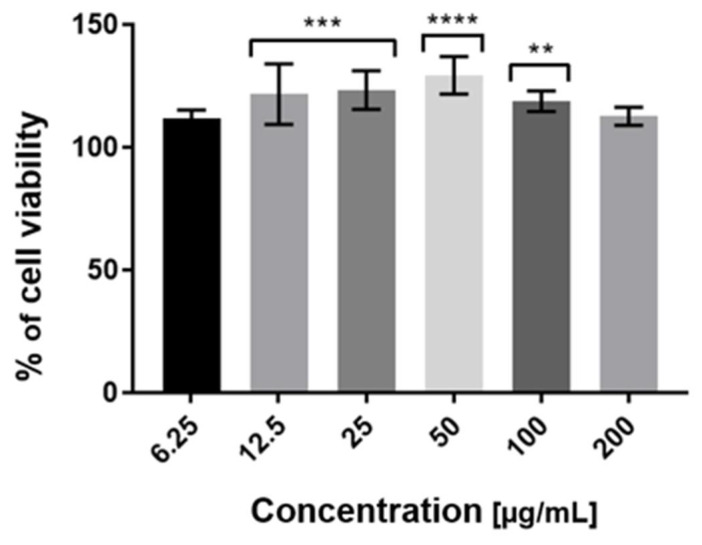
The reduction of resazurin after 24 h exposure to the aqueous MAE *Schisandra chinensis* extracts (6.75, 12.5, 25, 50, 100, 200 µg/mL) in cultured keratinocytes. Data are the mean ± SD of three independent experiments, each of which consists of three replicates per treatment group. ** *p* < 0.01, *** *p* < 0.001, **** *p* < 0.0001 versus the control (100%).

Our results obtained using the Alamar Blue assay showed that *Schisandra chinensis* extract in all tested concentrations (6.75–200 µg/mL) did not show a cytotoxic effect on HaCaT cells. Moreover, the induction of the proliferative effect of MAE extract was observed. The highest increase in cell proliferation, resulting from the largest number of active metabolic cells, was noticed for 50 µg/mL of tested extracts (Figure 5). Bearing in mind that resazurin reduction is the result of multiple metabolic reactions and it is not a direct indicator of mitochondrial dysfunction, we suppose that this bioassay is an appropriate tool for assessing cellular health and viability. Considering the observed tendency (for both assays), indicating that with increasing concentration of the extract above 50 µg/mL, cell proliferation decreases, it may be assumed that concentrations above 200 µg/mL may have cytotoxic effects on HaCaT cells.

### 2.4. Tyrosinase Activity

**Figure 6:**
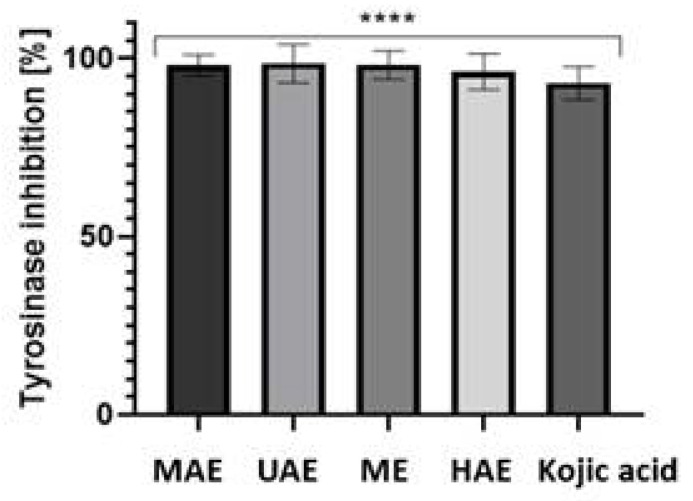
Inhibition of tyrosinase by four types of *Schisandra chinensis* fruits aqueous extracts (UAE-ultrasound-assisted extraction; MAE–microwave-assisted extraction; ME–maceration with continuous stirring; HAE–heat-assisted extraction). The concentration of the tested extracts was 50 µg/mL. Values are the mean of four replicate determinations (*n* = 4) ± SD. **** *p* < 0.0001 versus the control.

In our study, the antityrosinase effect of aqueous extract from *Schisandra chinensis* fruits was examined. The results of the performed experiments have shown that all four types of schisandra extracts are strong tyrosinase inhibitors. The percentage of inhibition ranged from 96.2 to 98.5%, depending on the type of extract (Figure 6). These results indicate that extracts from *S. chinensis* can be used as an anti-melanogenesis and skin-whitening agent. 

### 2.5. Irritant Potential of Model Body Wash Gels

**Figure 7:**
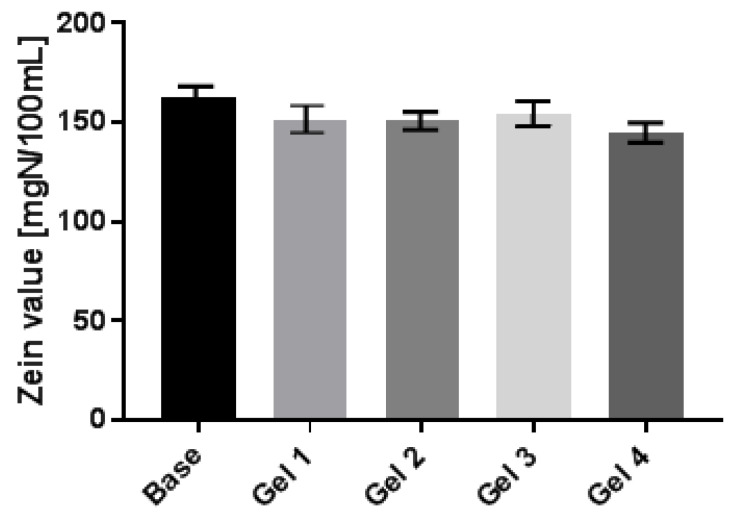
Zein value of model body wash gels containing water *S. chinensis* extracts. Four types of aqueous extract (UAE-ultrasound-assisted extraction (Gel 1); MAE–microwave-assisted extraction (Gel 2); ME–maceration with continuous stirring (Gel 3); HAE–heat-assisted extraction (Gel4)) are used. Model wash gels contain 3% (*w/w*) of appropriate extracts. The final values are the arithmetic mean of five independent measurements.

The results of the study indicate that the addition of all 4 tested schisandra extracts to body wash gels contributes to reducing the skin irritation potential of the products (Figure 7). Measurements of the zein number showed up to a 16% decrease in the irritant potential relative to the baseline sample. The greatest capacity to reduce the skin irritation effect was observed for the samples with the HAE aqueous *S. chinensis* extracts.

### 2.6. Rheological Properties of Model Body Wash Gels

**Figure 8:**
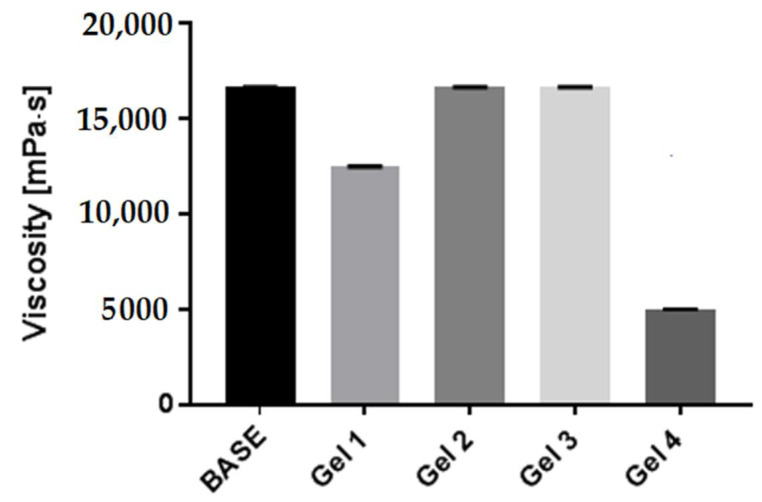
The viscosity of tested wash gel formulations containing aqueous *Schisandra chinensis* extracts was measured using a rheometer. Four types of aqueous extract (UAE-ultrasound-assisted extraction (Gel 1); MAE–microwave-assisted extraction (Gel 2); ME–maceration with continuous stirring (Gel 3); HAE–heat-assisted extraction (Gel4)) of *Schisandra chinensis* fruits are used. Values are the mean of six replicate determinations (*n* = 6).

In our study, it was observed that the addition of the *Schisandra chinensis* UAE and ME extracts did not change the viscosity of the model preparations compared to the baseline sample and remained at a similar level. However, the other two extracts (MAE and HAE) decreased the viscosity of natural body wash gel formulation containing the schisandra extracts in comparison with the extract-free baseline sample. The largest decrease in viscosity (ca. 4999 mPa·s) was observed for the sample containing the HAE extract (Figure 8). 

## 3. Discussion

Recent reports showed that *S. chinensis* fruit is a rich source of components with anti-inflammatory and antioxidant activity [9,18,21,22], which makes it a promising candidate for dermatological application. In our study, extract from the fruit was prepared using water to extrahent the safest for the skin and the extraction process was assisted by microwave, ultrasound and heating to increase the isolation of bioactive constituents. Generally, the phytochemical profile of the extracts was in line with the literature data [18,21,22,23]. Phenolic acids, flavonoids, anthocyanin and lignands were present in all water extracts; however, their quantity differed, and the efficacy of the extraction strongly depended on the technique used. Our results showed that microwave-assisted extraction was the most effective and MAE extract contained the highest content of flavonoids and anthocyanins compared to the other extract and the content of phenolic acid and lignans was similar to the extract obtained using elevated temperature. According to the literature, polyphenolic compounds found in the extract have valuable biological properties useful for cosmetology, such as anti-inflammatory, antibacterial, and antioxidant activity [17,23]. Some of them, including anthocyanins and quercetin-3-O-rutinoside (rutin), show sealing effects on capillaries, and protocatechuic acid is a strong inhibitor of lipid peroxidation [24]. Moreover, Schisandrol A–main lignan found in the extract shows estrogenic and proliferative potential [25], which may have significance in anti-aging products. It was also evidenced that lignins from Schisandra fruit displayed a dermo-protective effect on the skin exposed to smog conditions [11]. It suggests the synergistic action of multiple ingredients of schisandra fruit and makes it a valuable ingredient for cosmetic products [26].

An important property of phenolic compounds useful in cosmetology is an antioxidant effect which strongly depends on the chemical structure and correlates to the presence of hydroxyl groups and the positions of the groups in the molecular structure [27]. The antioxidant activity of polyphenols is based on diverse mechanisms, including, among others, the neutralization of free radicals through the stabilization or delocalization of the unpaired electron or the chelation of transition metal ions catalyzing reactions producing reactive oxygen species [28]. In our work, the antioxidant properties of Schisandra extracts were compared by analyzing the scavenging of the stable free radicals DPPH• and ABTS•+, and both tests showed a high antioxidant capacity of the extract correlated with phenolic content, and therefore, MAE extract occurred the most effective. 

In the further part of our study, the impact of the extract on HaCaT cells metabolism and viability was assessed using Neutral red (NR) uptake and Alamar Blue (AB) assay. NR relies on the ability to live cells to incorporate and bind neutral red dye in lysosomes. This cationic dye can penetrate cell membranes by nonionic passive diffusion and bind by electrostatic, hydrophobic bonds to anionic and phosphate groups of the lysosomal matrix [29]. The uptake of this dye depends on the cell’s capacity to maintain pH gradients, which is closely related to the production of ATP. At physiological pH, the dye presents a net charge close to zero, which allows it to penetrate the cell membranes. There is a proton gradient inside the lysosomes, which results in a lower pH than the pH of the cytoplasm. As a result, the dye becomes charged and is retained inside the lysosomes [30]. In turn, AB bioassay allows for the assessment of cell metabolic activity. This assay is based on fluorometric detection of the mitochondrial metabolic activity of the examined cells. This test allows us to measure changes in the fluorescence of the dye in the intracellular environment and detect the number of active metabolic cells [31].

The conducted analysis showed that the analyzed MAE extract did not cause a cytotoxic effect at any of the concentrations used. Numerous studies suggest a correlation between the content of biologically active compounds and a positive effect on cell viability in in vitro conditions. The compounds present in the tested extract, demonstrated in the course of spectrophotometric and chromatographic analyzes, have a positive effect on cell viability, proven in other studies. Phenolic compounds, including quercetin and kaempferol derivatives, play a positive role here, as they have antioxidant properties, the ability to reduce the level of free radicals in cells and exhibit cytoprotective and anti-apoptotic properties [32,33,34]. Additionally, other authors have shown that lignins present in *S. chinensis* extracts, such as deoxyschisandrin and Schizandrin B, can block Cox-2 cyclooxygenase, IL-6 interleukin and the IL-18 interleukin signaling pathway, thereby protecting skin cells from UVB radiation damage and greatly reduce the intracellular level of reactive oxygen species. Additionally, Schizandrin B may regulate the activity of superoxide dismutase (SOD), significantly reduce the production of many inflammatory factors, and lower the expression level of matrix metalloproteinase-1 (MMP-1), thus inhibiting the collagen degradation process [35,36,37,38].

Tyrosinase inhibition is another beneficial effect of *S. chinensis* extracts observed in our investigation. Tyrosinase is a copper-containing enzyme that plays a key role in melanin synthesis. It catalyzes two distinct reactions occurring during this process: the hydroxylation of tyrosine and the oxidation of 3,4-dihydroxyphenylalanine (L-DOPA) to o-dopaquinone. Abnormal and uncontrolled production and distribution of melanin can cause many dermatological disorders. Among melanin-related problems, we can distinguish lentigines, melasma and post-inflammatory hyperpigmentation [39,40]. To obtain new, efficient telomerase inhibitors, different types of compounds from natural sources have been intensively investigated. It is an important aspect in finding innovative approaches toward the regulation of the discoloration process [41]. Our study showed that all four types of schisandra extracts are strong tyrosinase inhibitors and the inhibition ranged from 96.2 to 98.5%. These results indicate that they can be applied as anti-melanogenesis and skin-whitening agents. The effect was also observed by other authors, both in the cell-free mushroom tyrosinase assay and cellular tyrosinase derived from cells cultivated in vitro [3,15].

Finally, the obtained extracts were used to prepare model gels. Cosmetic products are designed to integrate a variety of attributes, among which viscosity is a very important quality attribute. The viscosity measurements are performed to control factors associated with achieving desired application properties and making the cosmetic preparations more efficient. Due to their proven biological properties and rich in biologically active compounds, *S. chinensis* fruit extracts show beneficial effects on skin conditions. They are attributed to the following actions: toning, moisturizing or wound healing. Cosmetics containing the extracts cleanse and strengthen the skin barrier and soothe irritation [42]. In addition, studies have shown that Schisandrin B stimulates the production of glutathione, one of nature’s most potent antioxidants, which further supports the validity of the use of *S. chinensis* fruit extracts in anti-aging products [43,44].

## 4. Materials and Methods

### 4.1. Plant Material and Extraction Procedure

The fruits of *S. chinensis* were purchased from a local herbalist. It was produced by MyVita, Poland. The material was pulverized to an average particle size of about 0.3 mm. It was subsequently extracted with four methods detailed below.

#### 4.1.1. Ultrasound-Assisted Extraction Method (UAE)

Extracts from *Schisandra chinensis* fruits were obtained using the ultrasound-assisted extraction method (UAE). UAE was performed according to the method described by Yang et al. (2009) [45] in an ultrasonic bath (Digital Ultrasonic Cleaner) equipped with a time controller. 50 g of schizandra fruit was placed into a glass beaker, and 500 mL of water was poured. The mixture was sonicated at room temperature for 50 min (10 cycles for 5 min). Then, obtained extracts were collected and filtered three times through Whatman filter paper No. 10 using a vacuum pump. Extracts were stored in the dark at 4 °C for further analysis.

#### 4.1.2. Microwave-Assisted Extraction Method (MAE)

The microwave-assisted extraction was performed in a microwave oven (Tefal)according to the procedure described by Delazar et al. [46] with modifications. 50 g of schizandra fruit was placed in a Teflon vessel with 500 mL of water. Extraction was carried out for 50 min (10 cycles for 5 min) at 260 W with 10 s intervals. The obtained extract was filtered three times through Whatman filter paper No. 10 using a vacuum pump. The extract was stored in the dark at 4 °C for further analysis.

#### 4.1.3. Heat-Assisted Extraction Method (HAE)

In a glass beaker, 50 g of fruit was placed, and 500 mL of water was poured in. The mixture was stirred with a magnetic stirrer with a heating function for 24 h at 500 rpm at 50 degrees under the cover according to the procedure described by Antoniou et al. [47] with modifications. After extraction, the extract was filtered three times through Whatman filter paper No. 10 using a vacuum pump. The resulting suspension was stored in the dark at 4 °C.

#### 4.1.4. Maceration with Stirring (ME)

First, 50 g of fruit was placed in a glass beaker, and 500 mL of water was poured. The mixture was stirred with a magnetic stirrer for 24 h at 500 rpm according to the procedure described by Gori et al. [48] with modifications. Extraction was carried out at room temperature (about 22–24 °C). The obtained extract was filtered three times through Whatman filter paper No. 10 using a vacuum pump and stored in the dark at 4 °C for further analysis.

### 4.2. Total Phenolic Content Determination

The total phenolic content of *Schisandra chinensis* extracts was determined spectrophotometrically by the Folin-Ciocalteu method according to the procedure reported by Singleton et al. [49] with some modifications. The 300 μL of extract and 1500 μL of 1:10 Folin-Ciocalteau reagent were mixed. After 6 min of incubation in the dark, 1200 μL of sodium carbonate (7.5%) was added. After 2 h of incubation in the dark at room temperature, the absorbance of the test solutions was measured spectrophotometrically at λ = 740 nm by Aquamate Helion (Thermo Scientific, Waltham, MA, USA). Four extracts obtained using different extraction techniques were used in the measurements. The total phenolic concentration was calculated from a gallic acid (GA) calibration curve (10–100 mg/L). Data were expressed as gallic acid equivalents (GA)/100 g of material averaged from six measurements.

### 4.3. Total Flavonoids Content Determination

The total flavonoid content of *Schisandra chinensis* extracts was evaluated using aluminum nitrate nonahydrate according to the procedure reported by Majetic et al. [50]. The 600 μL of schizandra extracts solutions and 2400 μL of the mixture (80% C_2_H_5_OH, 10% Al(NO_3_)_3_·9 H_2_O and 1M C_2_H_3_KO_2_) were mixed. After 40 min of incubation at room temperature, the absorbance at 415 nm was measured spectrophotometrically by FilterMax F5 (Aquamate Helion). Four extracts obtained using different extraction techniques were used in the measurements. The total flavonoid concentration in tested extracts was calculated from a quercetin hydrate (Qu) calibration curve (10–100 mg/mL) and expressed as quercetin equivalents (Qu)/100 g of material averaged from six independent measurements.

### 4.4. UHPLC-MS Analysis

Analyses were carried out using RP18 reversed-phase column Titan (Supelco, Sigma-Aldrich, Burlington, MA, USA) (10 cm × 2.1 mm i.d., 1.9 µm particle size) on an ultra-high performance liquid chromatograph (UHPLC) Infnity Series II with a DAD detector and Agilent 6224 ESI/TOF mass detector (Agilent Technologies, Santa Clara, CA, USA). The thermostat temperature was 30 °C, and the flow rate was 0.2 mL/min. Water with 0.05% of formic acid (solvent A) and acetonitrile with 0.05% of formic acid (solvent B) was used as components of the mobile phase. The gradient elution program for flavonoids and phenolic acid was as follows: 0–8 min from 98% A to 93% A (from 2% to 7% B), 8–15 min from 93% A to 88% A (from 7% to 12% B), 15–29 min from 88% A to 85% A (from 12% to 15% B), 29–40 min from 85% A to 80% A (from 15% B to 20% B) and 40–60 min from 80% A to 65% A (from 20% B to 35% B). Gradient elution program for lignans was based on literature [22]: 0–10 min from 55% A to 52% A (from 45% to 48% B), 10–20 min from 52% A to 32% A (from 48% to 68% B), 20–25 min from 32% A to 22% A (from 68% to 78% B).

Chromatograms were in the range of 200–600 nm. LC–MS conditions: drying gas temperature 325 °C, drying gas flow 8 L min^−1^, nebulizer pressure 30 psi, capillary voltage 3500 V, and skimmer 65 V. The voltages on the fragmentator were: 180 V and 240 V. Ions were acquired from 100 to 1300 *m/z*. MS identification was done based on literature and comparison with standards. Quantification was based on calibration curves obtained using standard methanol solutions of identified compounds. All standards, formic acid and MS grade acetonitrile, were from Sigma-Aldrich (St. Louis, MO, USA).

### 4.5. Antioxidant Assay

#### 4.5.1. DPPH Radical Scavenging Assay

The free radical scavenging activity of *S. chinensis* extract was evaluated using DPPH (2,2-diphenyl-1-picrylhydrazyl) assay, according to the method described by Brand-Williams et al. [51]. 167 µL of 4 mM ethanol solution of DPPH was mixed with 33 µL analyzed samples in different concentrations (6.25, 12.5, 25, 50, 100, 200 µg/mL). Measurements were carried out for all four extraction methods. The absorbance was measured at λ = 516 nm every 5 min for 30 min using UV-Vis spectrophotometer Filter Max 5 (Thermo Scientific). DPPH solution mixed with an equal volume of distilled water served as a control. The percentage of the DPPH radical scavenging was calculated using Equation (1):

(1) $$\%\;\mathrm{DPPH}•\mathrm{scavenging}=\frac{\mathrm{Abs}\;\mathrm{control}-\mathrm{Abs}\;\mathrm{sample}}{\mathrm{Abs}\;\mathrm{control}}\times 100\%\;$$

#### 4.5.2. ABTS•+ Radical Scavenging Assay

An analysis of scavenging of the ABTS radical cation was performed according to the method described by Re et al. [52]. The scavenging reaction is based on the decolorization of the green ABTS radical cation (ABTS•+). To prepare the ABTS•+ solution, 19.5 mg ABTS and 3.3 mg potassium persulphate were mixed with 7 mL of phosphate buffer (pH = 7.4) and incubated in the dark for 16 h. In the next step, ABTS•+ solution was diluted to reach the absorbance at λ = 414 nm around 1.0. 20 µL portion of diluted extracts were added to 980 µL ABTS•+ solution and incubated at room temperature for 10 min. The absorbance was measured at λ = 414 nm using an Aquamate Helion spectrophotometer (Thermo Scientific). The distilled water was used as a blank. The percentage of the ABTS•+ scavenging was calculated using Equation (2):

(2) $$\%\;\mathrm{ABTS}•+\mathrm{scavenging}\;=1-\frac{\mathrm{Abs}\;\mathrm{sample}}{\mathrm{Abs}\;\mathrm{control}}\times 100\%\;$$

### 4.6. Tyrosinase Inhibitory Activity Measurement

In order to evaluate the inhibitory activity of tyrosinase by the *Schisandra chinensis* extracts, the procedure described by Studzińska-Sroka et al. [53] with L-DOPA as the substrate was used. Briefly, 80 μL of 50 mM phosphate buffer (pH 6.8) was added to each well containing 40 μL of extracts solution (UAE, SE, HAE and MAE) diluted in DMSO in a 96-well microtiter plate (VWR), then followed by 40 μL of mushroom tyrosinase solution (450 U/mL). The final concentration of the tested extracts was 4 mg/mL. After each well was mixed and pre-incubated at 25 °C for 10 min, 40 μL of 0.85 μM L-DOPA was added to the mixture and incubated at room temperature (25 °C) for 20 min. The absorbance of each well was measured at 492 nm by using a microtiter plate reader spectrophotometer FilterMax F5 (Thermo Fisher). Kojic acid was used as a positive control. The experiments were performed in four replications for each type of extract. The inhibitory effects of the test samples were expressed as the percentage of tyrosinase inhibition as follows (3):

(3) $$\%\;\mathrm{tyrosine}\;\mathrm{inhibition}=\frac{\left(A-B\right)-\left(C-D\right)}{A-B}\times 100\;\%\;$$

where: 

A—the optical density of the mixture without test sample, 

B—the optical density of the mixture without test sample and enzyme,

C—the optical density of the mixture with test sample and enzyme, 

D—the optical density of the mixture without enzyme.

### 4.7. Cell Culture

HaCaT (normal human keratinocytes) was obtained from the American Type Culture Collection (Manassas, VA 20108, USA). HaCaT cells were maintained in a DMEM (Dulbecco’s modified essential medium, Gibco) supplemented with L-glutamine, 5% (*v*/*v*) FBS (fetal bovine serum, Gibco), and 1% (*v/v*) antibiotic (100 U/mL Penicillin and 1000 µg/mL Streptomycin, Gibco). Cells were maintained at 37 °C in a humidified atmosphere of 95% air and 5% of carbon dioxide (CO_2_). When the cells reached confluence, the culture medium was removed from the flask (VWR), and cells were rinsed two times with sterile PBS (Phosphate-Buffered Saline, Gibco). The confluent layer was trypsinized using Trypsin/EDTA (Gibco) and then resuspended in a fresh medium. Cells were treated with varying concentrations (6.25, 12.5, 25, 50, 100, 200 µg/mL) of schizandra MAE extract suspended in the culture medium. The MAE extract was chosen because of the high total phenolic and flavonoid content and high free radical scavenging activity.

### 4.8. Cell Viability Assay

#### 4.8.1. Neutral Red Uptake Assay

Cell growth was measured using the neutral red dye (Sigma Aldrich, St. Louis, MO, USA). This assay is based on the initial protocol described by Borenfreund et al. [54]. HaCaT cells were placed in 96-well plates at a density of 1 × 10^4^ cells/well with fresh medium. After 24 h of pre-culture, the medium was aspirated and tested concentrations of *Schisandra chinensis* MAE extracts (6.25–200 µg/mL) were added into each well and cultured for another 24 h. The control group was unexposed cells. Following exposure to tested extracts, cells were incubated for 2 h with neutral red dye (40 µg/mL) dissolved in serum-free DMEM medium. Then, cells were washed with Phosphate Buffered Saline (PBS) and then added 150 µL destain solution (EtOH/AcCOOH/H_2_O_2_, 50%/1%/49%) per well, followed by gentle shaking for 10 min until the neutral red had been extracted from the cells and has formed a homogenous solution. Neutral red dye uptake was determined by measuring the optical density (OD) of the eluted dye at 540 nm in a microtiter plate reader spectrophotometer FilterMax F5 (Thermo Fisher). The experiments were performed in triplicates for each extract concentration and presented as a percentage of control values (100%).

#### 4.8.2. Alamar Blue Assay

The resazurin sodium salt (Alamar Blue) (Sigma, R7017) was used to assess HaCaT cell viability according to the procedure by Page et al. [55]. Cells were seeded in transparent 96- well plates at a density of 1 × 10^4^ cells/well with fresh DMEM medium and exposed to different concentrations (6.25–200 µg/mL) of tested *Schisandra chinensis* MAE extracts for 24 h. The control group was unexposed cells maintained in a DMEM medium. After exposure, the resazurin solution was transferred into the plates for a final volume of 250 µL/well and the final concentration of 60 µM resazurin. Then, the cells were incubated for 2 h at 37 °C in darkness. The absorbance was measured at the wavelength λ = 570 nm using a microplate reader (FilterMax F5, Molecular Devices). The experiments were performed in triplicates for each *Schisandra* extract concentration. Results were expressed as percentage cell viability versus the control (100%).

### 4.9. Technology for Obtaining Prototypical Body Wash Gels

Based on prior experiences, prototypical body wash gel formulations containing the previously prepared four types of aqueous *Schisandra chinensis* extracts (at a concentration of 4 mg/mL) were developed. The final concentration of the extract in the model formulations was 280 µg/mL. The formulations meet the requirements applicable to natural cosmetics. The composition of prototypical natural body wash gel formulations is listed in Table 4.

In our research, five samples of prototypical body wash gels were obtained. The preparation method was the same for all the model samples. In the beginning, demineralized water and a preservative (Sodium Benzoate and Potassium Sorbate) were poured into a beaker. The contents of the beaker were heated to the temperature of 60 °C. In the next step, the washing agents (Lauryl Glucoside, Cocamidopropyl Betaine, Cocamide DEA, PEG-75 Lanolin) were added successively. All ingredients in the beaker were thoroughly mixed until the substances dissolved completely, using a magnetic stirrer (propeller mixer, speed 250 rpm). To modify the viscosity of the formulations, Lactic Acid was added. Then, samples were cooled, and the previously prepared aqueous Schisandra chinensis extracts were added. Four types of aqueous extract (UAE (Gel 1), MAE (Gel 2), ME (Gel 3), and HAE (Gel4)) were used. An extract-free sample (Base) was used as a reference sample. Each of the prototypical body wash gels was homogenized (IKA homogenizer; 3000 rpm) and stored at room temperature until complete air elimination.

### 4.10. Zein Test

The irritant potential of the natural body wash gel formulation containing the previously prepared *S. chinensis* extracts was measured using a zein test. In the surfactants solution, zein protein is denatured and subsequently solubilized in the solution. This process simulates the behavior of surfactants in relation to the skin proteins. 2 g of zein from corn was added to 40 mL of sample solution (10%). The mixture was shaken on a shaker with a water bath (60 min at 37 °C). Subsequently, the tested solutions were filtered on Whatman No. 1 filters and centrifuged at 6720 g for 10 min. The nitrogen content in the solutions was determined by the Kjeldahl method. Initially, 1 mL of the filtrate was mineralized in sulfuric acid (98%) containing copper sulphate pentahydrate and potassium sulphate. Then, the solution was transferred (with 50 mL of MiliQ water) into the flask of the Wagner–Parnas apparatus. 20 mL of sodium hydroxide (25%) was added. In the next step, the released ammonia was distilled with steam and bound by sulfuric acid (5 mL of 0.1 N H_2_SO_4_) in the receiver of the Wagner–Parnas apparatus [56]. The unbound sulfuric acid was titrated with 0.1 N sodium hydroxide. Tashiro solution was used as an indicator. The zein number (ZN) was calculated using Equation (4):

(4) $$\mathrm{Zein}\;\mathrm{number}=\left(10-\;V1\right)\times 100\times 0.7$$

where V1 is the volume (mL) of sodium hydroxide used for titration of the sample. The final result was the arithmetic mean of five independent measurements.

### 4.11. Viscosity Measurements

The viscosity of the tested wash gel formulations was measured using a Fungilab Expert (Fungilab, Barcelona, Spain) rheometer. Measurements were carried out at 22 °C with a rotary speed of the spindle of 10 rpm [57]. Viscosity values presented in the figures represent average values obtained from six independent measurements.

### 4.12. Statistical Analysis

The points in the charts represent mean values from a series of three, five or six independent measurements. Values of different parameters were expressed as the mean ± standard deviation (SD). The one-way analysis of variance (ANOVA) and Bonferroni posttest between groups was performed at the level *p*-value of <0.05 to evaluate the significance of differences between values. Statistical analyses were performed using GraphPad Prism 5.0 (GraphPad Software, Inc., San Diego, CA, USA).

## 5. Conclusions

The work attempts to determine the properties and the possibility of using four types of *Schisandra chinensis* water extracts in skin care products. The obtained results indicate that the tested extracts are characterized by a high content of phenolic compounds and flavonoids and show high antioxidant potential. In addition, these extracts positively affect the viability and metabolic activity of keratinocytes and strongly inhibit tyrosinase activity, which can significantly inhibit hyperpigmentation and be helpful in the treatment of skin hyperpigmentation disorders. Studies have also shown that the addition of these extracts to body wash gel preparations has a positive effect on their quality and can help reduce skin irritation. Moreover, the results obtained as part of this work indicate a noticeable effect of the extraction method used, which affects the amount of biologically active compounds in the obtained extracts, thus significantly affecting their properties. In addition, the lack of a cytotoxic effect on skin cells in vitro and the possibility of reducing the irritating effect of cosmetic preparations may suggest a relatively high safety of extracts obtained from this plant material and indicate its potential use in a wide range of preparations for the care and treatment of various skin diseases. To determine the exact effect of this plant, additional research is needed, but the results obtained in this work suggest that extracts from *Schisandra chinensis* fruit can be perceived as a multi-directional raw material used in the cosmetic and pharmaceutical industries.

## Figures and Tables

**Table 1:** MS data of polyphenolic components identified in *S. chinensis* fruits extract at negative ionization mode.

T_R_ (min.)	Observed Ion Mass [M-H]-/(Fragments)	Δppm	Formula	Identified *	Ref.
1.56	191.05701	4.68	C_7_H_12_O_6_	quinic acid	str
8.01	153.01946 (109)	0.83	C_7_H_6_O_4_	protocatechuic acid	str
10.37	353.08872 (179)	2.58	C_16_H_18_O_9_	neochlorogenic acid	[18], str
13.33	337.09293 (163)	0.12	C_16_H_18_O_8_	3-p-coumaryl quinic acid	[18]
15.52	353.08895 (179)	3.23	C_16_H_18_O_9_	chlorogenic acid	[18], str
18.51	337.09457 (173)	4.97	C_16_H_18_O_8_	4-p-coumaryl quinic acid	[18]
19.37	337.09338 (191)	1.45	C_16_H_18_O_8_	5-p-coumaryl quinic acid	[18]
31.72	609.14762 (300,463)	2.48	C_27_H_30_O_16_	quercetin-3-*O*-rutinoside	[18], str
32.17	463.08881 (300)	1.32	C_21_H_20_O_12_	quercetin-3-*O*-galactoside	[18]
32.75	463.08864 (300)	0.95	C_21_H_20_O_12_	quercetin-3-*O*-glucoside	[18], str
37.48	593.15299 (285)	3.02	C_27_H_30_O_15_	kaempferol-3-*O*-rutinoside	[18], str
38.30	447.09354 (285)	0.57	C_21_H_20_O_11_	kaempferol-3-*O*-glucoside	[18], str

* compounds were tentatively identified based on mass data when the standard was not available (str- standard).

**Table 3:** The concentration of phenolic acids and flavonoids (µg/mL) in *S. chinensis* fruit extracts was obtained using microwave (MAE), ultrasound (UAE), heat extraction (HAE) and maceration with continuous stirring (ME).

	MAE	UAE	HAE	ME
Phenolics acids				
Protocatechuic	2.07 ± 0.11	0.92 ± 0.04	1.65 ± 0.07	1.07 ± 0.08
Chlorogenic (total)	0.22 ± 0.02	nd	0.18 ± 0.01	nd
p-coumaryl quinic ^1^ (total)	11.53 ± 0.52	6.31 ± 0.32	12.04 ± 0.81	8.49 ± 0.54
Flavonoids				
Quercetin-3-*O*-rutinoside	0.58 ± 0.03	0.22 ± 0.01	0.46 ± 0.03	0.37 ± 0.03
Quercetin-3-*O*-galactoside ^2^	0.42 ± 0.02	0.19 ± 0.02	0.31 ± 0.02	0.23 ± 0.02
Quercetin-3-*O*-glucoside	0.85 ± 0.03	0.22 ± 0.02	0.47 ± 0.03	0.21 ± 0.02
Kaempferol-3-*O*-rutinoside	0.98 ± 0.04	0.27 ± 0.01	0.70 ± 0.04	0.35 ± 0.02
Kaempferol-3-*O*-glucoside	0.54 ± 0.04	0.24 ± 0.02	0.54 ± 0.02	0.14 ± 0.01
Anthocyanin				
cyanidin 3-*O*-xylosyl-rutinoside ^3^	0.18 ± 0.02	0.10 ± 0.01	0.15 ± 0.02	0.14 ± 0.02

Quantification was based on calibration curves for ^1^ *p*-coumaric acid, ^2^ Quercetin-3-*O*-glucoside, and ^3^ cyanidin 3-*O*-glucoside; nd-not detected.

**Table 4:** Formulation of model body wash gels.

Ingredient (INCI)	Ingredient Content (%)
Aqua	to 100.0
Lauryl Glucoside	7.0
*Schisandra chinensis* extract	3.0
Cocamidopropyl Betaine	5.0
Cocamide DEA	1.5
Sodium Benzoate and Potassium Sorbate	1.0
PEG-75 Lanolin	0.5
Lactic Acid	0.3

## Data Availability

Data are contained within the article.

## Supplementary Materials

The following supporting information can be downloaded at: https://www.mdpi.com/article/10.3390/molecules27248877/s1, Figure S1: Representative chromatograms of MAE *S. chinensis* fruits extract including total ion chromatogram (negative mode) and chromatograms recorded at 254 and 320 nm.; Figure S2: Representative chromatograms of lignans in MAE *S. chinensis* fruits extract, including total ion chromatogram (positive mode) and chromatogram recorded at 254. Figure S3: Representative MS spectra of lignans found in *S. chinensis* fruits extract; Figure S4: Representative UV-Vis spectra of compounds identified in extracts of *Schisandra chinensis* fruits; Figure S5: Comparison of lignans content (signal intensity) between four types of aqueous extracts of *Schisandra chinensis* fruits.
