# Supplementary material for: Evaluation of the Antioxidant, Cytoprotective and Antityrosinase Effects of Schisandra chinensis Extracts and Their Applicability in Skin Care Product
Source: Molecules. 2022 Dec 14;27(24):8877. doi: 10.3390/molecules27248877 (PMC9786222; doi:10.3390/molecules27248877)
Supplement: Supplementary file 1 [file molecules-27-08877-s001.zip › molecules-2045013-supplementary.pdf]

molecules

Supplementary material

Evaluation of the antioxidant, cytoprotective and antityrosinase effects of *Schisandra chinensis* extracts and their applicability in skin care product

Martyna Zagórska-Dziok[1], Magdalena Wójciak[2*], Aleksandra Ziemlewska[1], Zofia Nizioł-Łukaszewska[1], Uliana Hoian[1], Katarzyna Klimczak[1], Dariusz Szczepanek[3], Ireneusz Sowa[2]

[1] Department of Technology of Cosmetic and Pharmaceutical Products, Medical College, University of Information Technology and Management in Rzeszow, Kielnarowa 386a, 36-020 Tyczyn, Poland
[2] Department of Analytical Chemistry, Medical University of Lublin, Chodźki 4a, 20-093 Lublin, Poland[3] Chair and Department of Neurosurgery and Paediatric Neurosurgery, Medical University of Lublin, 20-090 Lublin, Poland; dariusz.szczepanek@umlub.pl (D.S.)

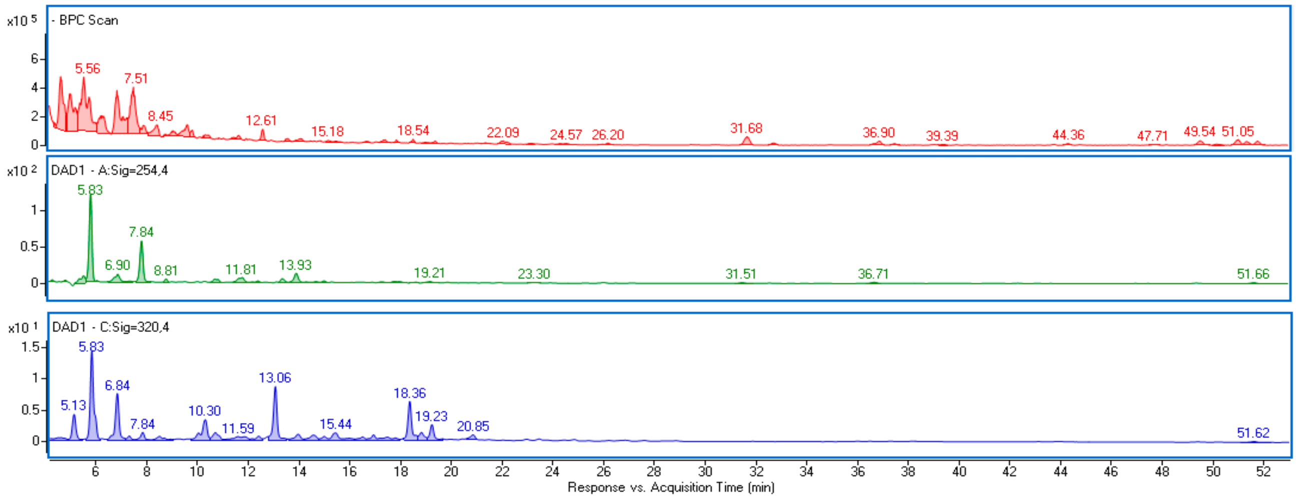

Figure S1. Representative chromatograms of MAE *S. chinensis fruits* extract including total ion chromatogram (negative mode) and chromatograms recorded at 254 and 320 nm.

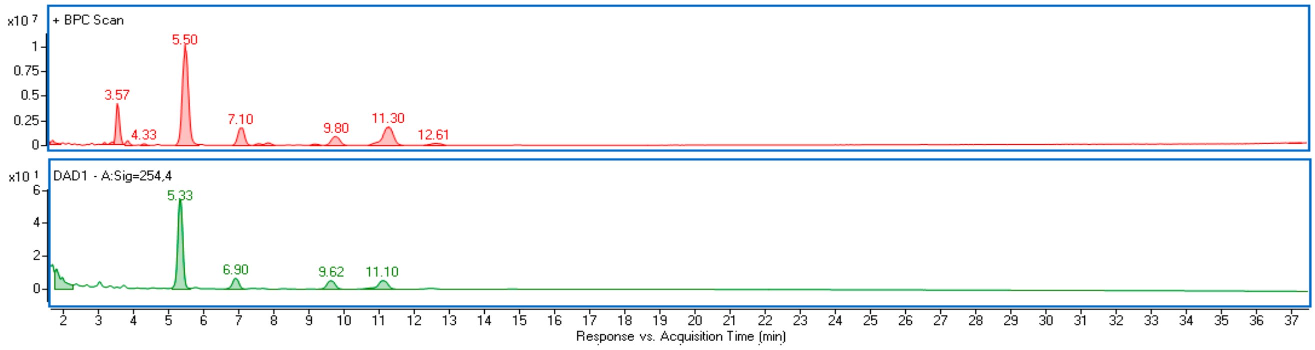

Figure S2. Representative chromatograms of lignans in MAE *S. chinensis fruits* extract including total ion chromatogram (positive mode) and chromatogram recorded at 254.

.

(a)

(b)

(c)

(d)

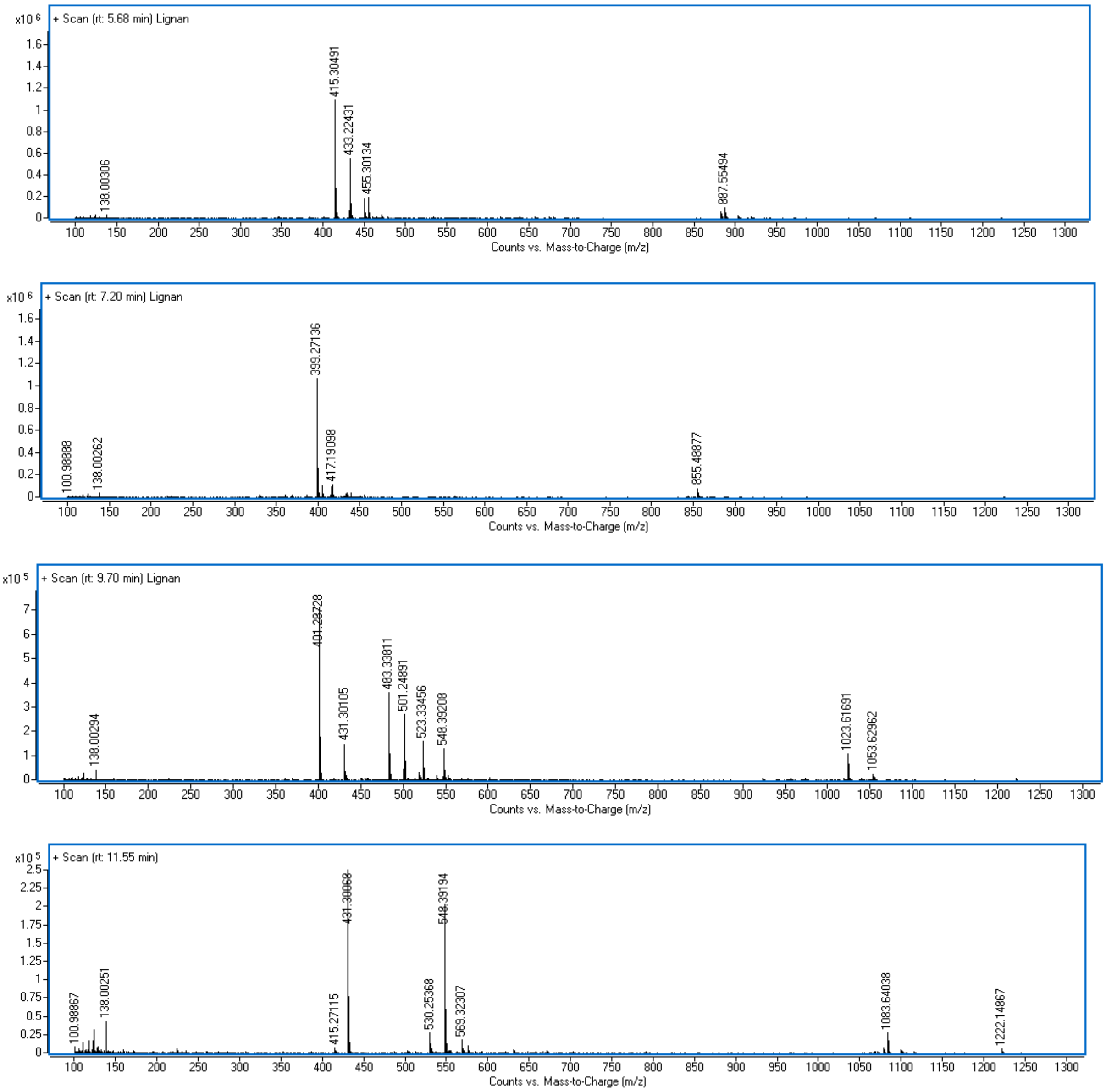

Figure S3. Representative MS spectra of lignans found in *S. chinensis fruits* extract: (a) Schisandrol A, (b) Schisandrol B, (c) Micrantherin A, (d) Angeloylgomisin Q.

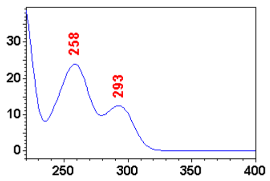

protocatechuic acid

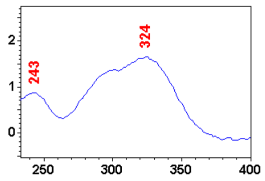

neochlorogenic acid

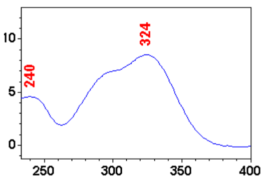

chlorogenic acid

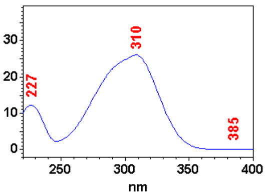

3-p-coumaryl quinic acid

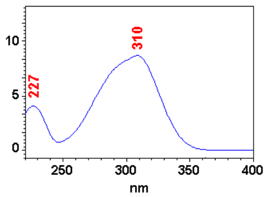

4- p-coumaryl quinic acid

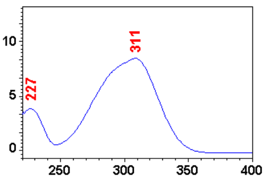

5- p-coumaryl quinic acid

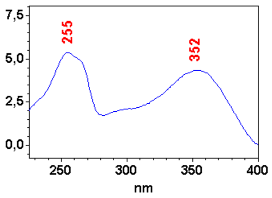

quercetin -3-O-galactoside

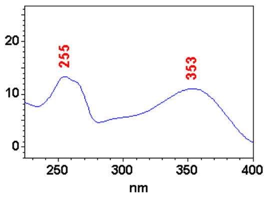

quercetin-3-O-rutinoside

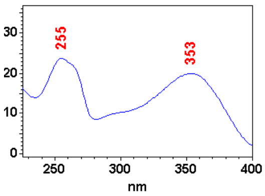

quercetin-3-O-glucoside

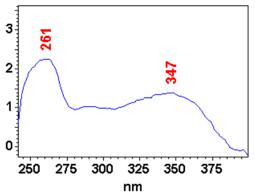

kaempferol-3-O-rutinoside

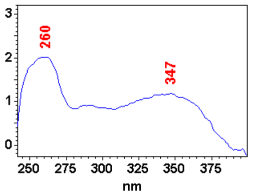

kaempferol-3- O-glucoside

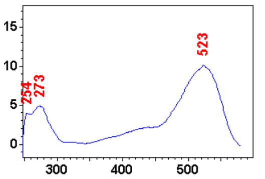

cyanidin
3-O-xylosyl-rutinoside

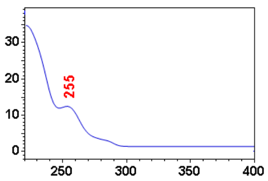

Schisandrol A

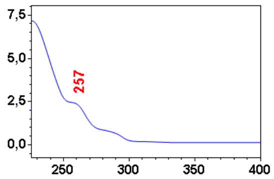

Schisandrol B

Figure S4. Representative UV-Vis spectra of compounds identified in extracts of *Schisandra chinensis* fruits.

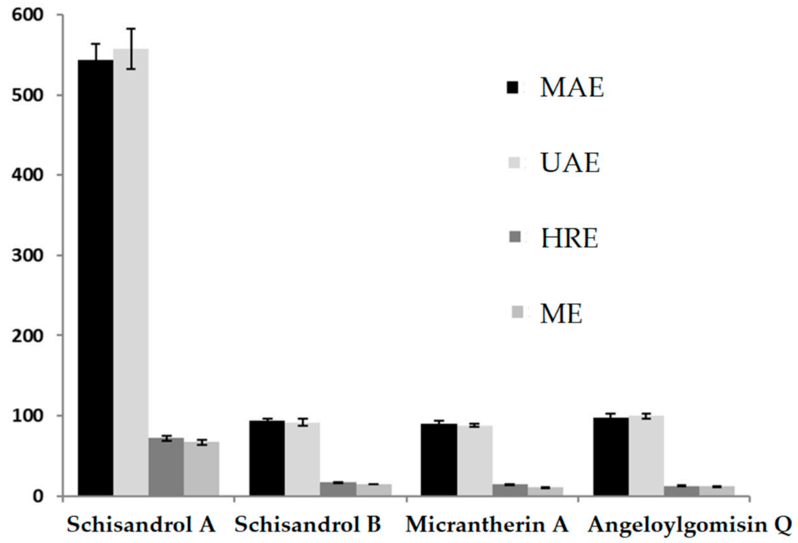

Figure S5. Comparison of lignans content (signal intensity) between four types of aqueous extracts of *Schisandra chinensis* fruits: UAE -ultrasound-assisted extraction; MAE - microwave-assisted extraction; ME - maceration with continuous stirring; HAE - heat-assisted extraction. Values are mean of three replicate determinations (n=3) ± SD.
